# Myeloperoxidase as a biomarker for intestinal-brain axis dysfunction induced by malnutrition and *Cryptosporidium* infection in weanling mice

**DOI:** 10.1016/j.bjid.2023.102776

**Published:** 2023-05-05

**Authors:** Reinaldo B. Oriá, Deiziane V.S. Costa, Pedro Henrique Q.S. de Medeiros, Cássia R. Roque, Ronaldo P. Dias, Cirle A. Warren, David T. Bolick, Richard L. Guerrant

**Affiliations:** a Faculdade de Medicina da Universidade Federal do Ceará, Departamento de Morfologia e Instituto de Biomedicina, Laboratório de Cicatrização de Tecidos, Ontogenia e Nutrição, Fortaleza, CE, Brazil; b University of Virginia School of Medicine, Department of Medicine, Division of Infectious Diseases and International Health, Center for Global Health Equality, Charlottesville, USA; c Faculdade de Medicina da Universidade Federal do Ceará, Instituto de Biomedicina, Laboratório de Doenças Infecciosas, Fortaleza, CE, Brazil

**Keywords:** *Cryptosporidium parvum*, Malnutrition, Gut, Neuroinflammation, Myeloperoxidase

## Abstract

Cryptosporidiosis is a waterborne protozoal infection that may cause life-threatening diarrhea in undernourished children living in unsanitary environments. The aim of this study is to identify new biomarkers that may be related to gut-brain axis dysfunction in children suffering from the malnutrition/infection vicious cycle is necessary for better intervention strategies. Myeloperoxidase (MPO) is a well-known neutrophil-related tissue factor released during enteropathy that could drive gut-derived brain inflammation. We utilized a model of environmental enteropathy in C57BL/6 weanling mice challenged by *Cryptosporidium* and undernutrition. Mice were fed a 2%-Protein Diet (dPD) for eight days and orally infected with 10^7^-*C. parvum* oocysts. *C. parvum oocyst* shedding was assessed from fecal and ileal-extracted genomic DNA by qRT-PCR. Ileal histopathology scores were assessed for intestinal inflammation. Prefrontal cortex samples were snap-frozen for MPO ELISA assay and NF-kb immunostaining. Blood samples were drawn by cardiac puncture after anesthesia and sera were obtained for serum amyloid A (SAA) and MPO analysis. Brain samples were also obtained for Iba-1 prefrontal cortex immunostaining. *C. parvum*-infected mice showed sustained stool oocyst shedding for six days post-infection and increased fecal MPO and inflammation scores. dPD and cryptosporidiosis led to impaired growth and weight gain. *C. parvum*-infected dPD mice showed increased serum MPO and serum amyloid A (SAA) levels, markers of systemic inflammation. dPD-infected mice showed greater MPO, NF-kB expression, and Iba-1 immunolabeling in the prefrontal cortex, an important brain region involved in executive function. Our findings suggest MPO as a potential biomarker for intestinal-brain axis dysfunction due to environmental enteropathy.

## Introduction

Cryptosporidiosis is a water-borne protozoa infection transmitted by fecal-oral contact, which has been associated with life-threatening chronic diarrhea in immune-compromised individuals^1^^,^^2^ and with growth faltering in children (even without overt diarrhea) living in unsanitary settings of the developing world.^3^ More recently, *Cryptosporidium* was listed among the leading causes of moderate-to-severe diarrhea under five years old from seven different sites from sub-Saharan Africa and South Asia.^4^

The long-term impact of enteric infections, including cryptosporidiosis, on children's development has gained more interest,^5^ as the first two years of life are a critical time with profound changes in synaptogenesis, myelination, and brain circuitry modeling in the child's brain, which could compromise one's full cognitive potential.^6^ There is now growing evidence from large multi-center birth cohorts that enteric infections and undernutrition during child development may contribute to cognitive deficits even later in life,^7^, ^8^, ^9^ thus confirming an early study reporting an association between cryptosporidial infections and impaired cognition in children from northeast Brazil.^10^ Nevertheless, the mechanistic links between enteropathy, the vicious cycle of undernutrition-enteric infections, and neurocognitive alterations are not clearly understood.

In the context of aging-related brain injury, pre-clinical studies have been describing the role of myeloperoxidase (MPO) in neurodegenerative diseases, such as Alzheimer's disease,^11^ Parkinson's disease,^12^ and multiple sclerosis.^13^ However, potential gut-driven mechanisms (and biomarkers) of neuroinflammation induced by enteric infections and undernutrition remain elusive. Such biomarkers could identify children who are at greater at-risk of intestinal-brain axis dysfunction and cognitive impairment for better and earlier interventions.

To explore the potential interactions of enteropathy and/or *Cryptosporidium* infections with neuroinflammation, we used a weaned mouse model of protein-deficient diet and *C. parvum* infection, which leads to marked growth impairment, parasite burden, and intestinal/systemic inflammation, thus providing a reproducible model of environmental enteropathy.^14^^,^^15^ In this model, we assessed gut, blood and prefrontal cortex MPO, a well-known neutrophil-related inflammatory factor, that is released during enteropathy,^16^ as a target biomarker for gut-driven brain inflammation. Further, we measured other inflammatory mediators in the brain, NF-kB expression, and ionized calcium-binding adaptor-1 (Iba-1) expression.

## Material and methods

### Mice, diets, and Cryptosporidium infection model

In our study, we used 3-old week C57BL/6J male mice (a total of 61) that were purchased from Charles River Laboratories (Wilmington, MA, USA). Experimental weanling mice received all diets and water *ad libitum* for 2 weeks until the endpoint. Nourished mice (a total of 22 mice, *n* = 10 per experiment) received a chow diet (defined nutrition diet, dND). To induce undernourishment, mice received a protein deficient diet containing 2% of protein (dPD) (a total of 20 mice, *n* = 10 per experiment). All diets were from Envigo, Teklad, VA, USA. Composition of the experimental diets is described elsewhere.^17^ Bodyweight was measured every 3 days. On the 9th day, the animals were infected with *C. parvum* (a total of 10^7^ unexcysted oocysts) (a total of 19 mice, *n* = 10 per experiment). Over the following 6 days, daily measurements of body weight and collection of feces were performed. Euthanasia was done by cervical dislocation after CO_2_ overdose. After infection, stools were assessed for *C. parvum* oocyst shedding and inflammatory biomarkers. Tail and body length was recorded immediately after euthanasia as markers of skeletal growth. Protocols from this study were previously approved by the Institutional Animal Care and Use Committee at the University of Virginia.

### Inoculum preparation

Preparation and administration of unexcysted *C. parvum* oocysts were as described elsewhere.^18^ Each infected mouse received 100 µL of PBS plus freshly prepared unexcysted *C. parvum* oocysts in a recently vortexed solution (10^7^-oocysts per mouse) by oral gavage directly into the stomach. Control mice received 100 µL of PBS by oral gavage at the same time.

### Analysis of *C. parvum* oocyst shedding in stools

Analyses by qRT-PCR with fecal and ileal *C. parvum* oocyst shedding were assessed by genomic DNA extracted from fecal and ileal samples. The primers target the 18 s rRNA gene of the parasite by GenBank (www.ncbi.nlm.nih.gov/genbank/, AF164102). The reaction was performed in a Bio-Rad iCycler iQ multicolor PCR Detection System using iCycler software (version 3.0). Amplification consisted of 15 min at 95 °C followed by 40 cycles of 15 s at 95 °C, 15 s at 52 °C, and 20 s at 72 °C, followed by 0.5-degree increments for 10 s starting at 75 °C and ending with 95 °C for the Melt Curve. Ct values of each run were compared to standards with known amounts of *C. parvum* DNA and log transformed into number of organisms per mg of stool sample.^19^

### Measurement of microscopic damage

Mice ileum tissues from day 7 p.i. were fixed in 10% neutral buffered formalin for 20 h, dehydrated and embedded in paraffin. Ileum sections (5 µm) were then stained with Hematoxylin and Eosin staining (H&E) and examined using light microscopy. Histopathological scores were performed by a blinded investigator, using a previously described method^20^ with some modifications. Histopathological scores were determined by quantifying the intensity of epithelial tissue damage (0‒3, 0 - No damage, 1 - Mild, 2 - Moderate, 3 - Extensive), and cell infiltration (0‒3). The total histological damage score was measured by the sum of the two parameters evaluated.

### Measurement of myeloperoxidase and serum amyloid A levels

Prefrontal cortex samples were snap-frozen in liquid nitrogen for myeloperoxidase (MPO) measurement. At the time of assay, samples were lysed in RIPA buffer (20 mM Tris, Ph 7.5, 150 mM NaCl, 1% Nonidet P-40, 0.5% sodium deoxycholate, 1 mM EDTA, 0.1% SDS), containing protease inhibitor cocktail (Roche of Millipore Sigma, St Louis, MO, USA). Tissue lysates were cleared by centrifugation at 8000× g for 10 min at rt, and the supernatant fluid was collected. Specific DuoSet® ELISA kit (R&D Systems, Minneapolis, MN, USA) was used for MPO analyses according to manufacturer's instructions.

Blood samples were drawn by cardiac puncture after light anesthesia with ketamine/xylazine solution. Serum was obtained and immediately frozen and stored in −20° freezer until analyses. Serum Amyloid A (SAA) and MPO were analyzed as markers of systemic inflammation, according to specific DuoSet® ELISA kit (R&D Systems, Minneapolis, MN, USA).

### Iba-1 immunostaining

Iba-1 prefrontal cortex immunostaining was adapted from Mandwie et al.^21^ In brief, brain samples were obtained from saline perfused animals followed by paraformaldehyde (PFA; ∼20 mL). 20 µm sections of the brain were cut in the cryostat and carefully transferred to a 96-well plate with PBS. Coronal sections were incubated overnight with mouse IgG-Iba-1 primary antibodies (1:500 in blocking solution/normal goat serum; Santa Cruz Biotechnology, Dallas, TX, USA) at 4 °C, washed three times in PBS for 5 min, and incubated with Alexa Fluor 488-conjugated goat anti-mouse secondary antibody. DAPI was used to determine the number of nuclei. Finally, the sections were mounted onto Fisherbrand® Superfrost plus microscopic slides (Fisher Scientific, Pittsburgh, PA, USA) and coverslipped. Fluorescent images were obtained on a FV-1000 Olympus Laser Scanning Microscope (Shinjuku, Tokyo, Japan).

### Western blotting for NF-kB

NF-kB protein assay was adapted from Wang et al.^22^ Prefrontal cortices were processed for western blotting according to standard procedures. Following membrane blocking with BSA (5%), samples were incubated with primary antibodies (rabbit anti-NF-KB, 1:500 overnight at 4 °C, followed by incubation with alkaline phosphatase-conjugated secondary antibody anti-rabbit IgG (1:1000). Internal control for protein content in each sample was evaluated by mouse anti-β-actin (1:1000). All antibodies were from Santa Cruz Biotechnology (Dallas, TX, USA). Targeted protein bands were visualized following membrane incubation with enhanced chemifluorescence using a ChemiDoc Imaging System (Biorad, Hercules, CA, USA) and quantified using Image Lab software (Biorad).

### Statistical analysis

The data are presented as the mean ± standard error of the mean (SEM). Student's *t*-test or one- or two-way analysis of variance (ANOVA) followed by the Turkey test was used to compare means; *p* < 0.05 was considered to indicate significance.

## Results

**Fig. 1:**
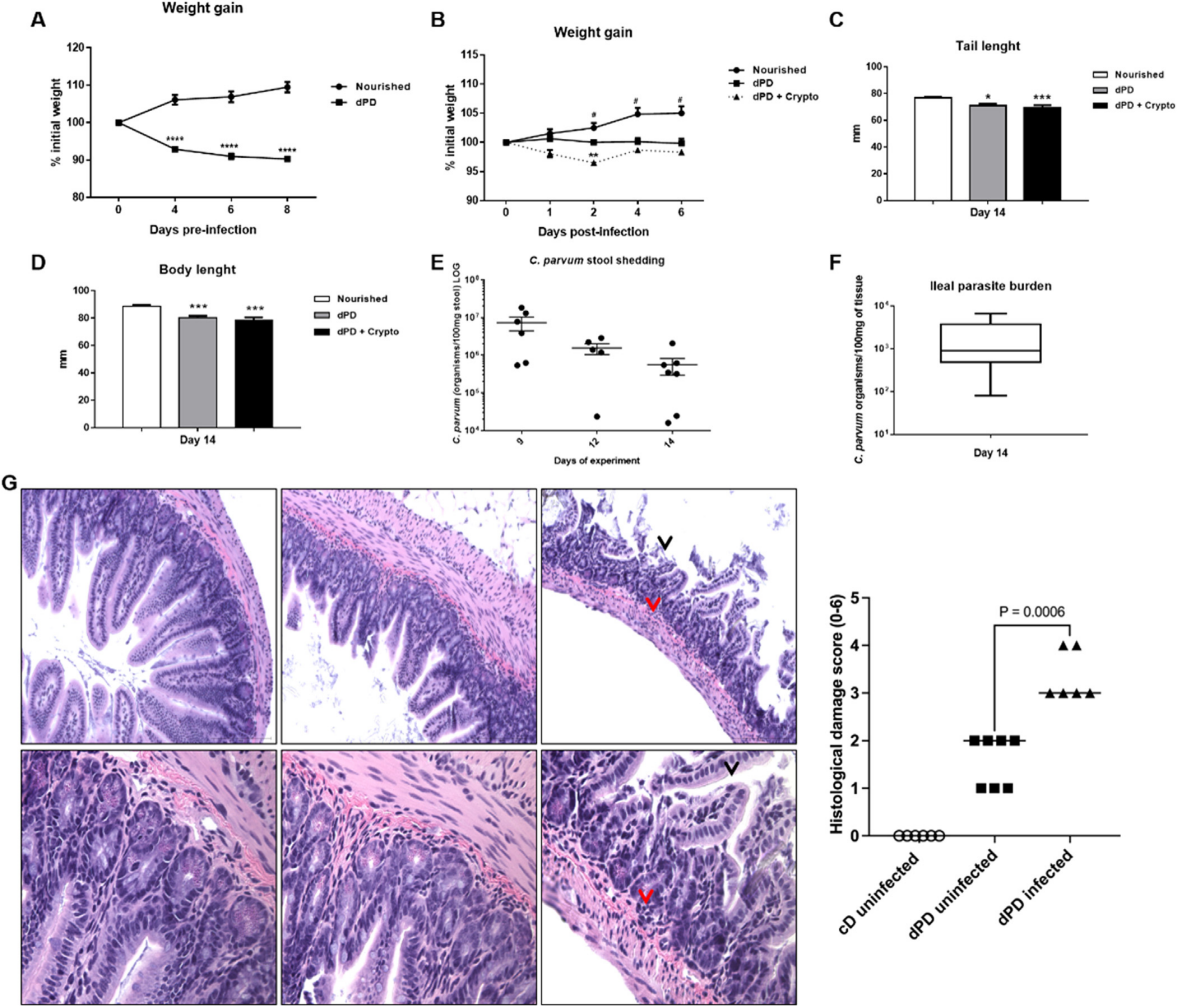
Cryptosporidium colonizes and induces impairment of growth in protein-deficient mice. (A) Percentual of body weight loss (mean ± SEM) of C57BL/6 mice receiving a nourished diet (*N* = 10) or protein-deficient diet (dPD) (*N* = 10) before *C. parvum* infection. (B) Percentual of body weight loss (mean ± SEM) of C57BL/6 mice receiving a nourished diet (*n* = 10) or protein-deficient diet (dPD) (*N* = 10) after *Cryptosporidium* infection. *p < 0.05, **p < 0.01 and ****p < 0.0001. (C) Tail and (D) body length (mean ± SEM) of uninfected (nourished or dPD; *N* = 9) and infected mice (dPD + Crypto; *N* = 9) on day 14 of the experimental protocol. *p < 0.05 and ***p < 0.01. (E) Quantification of *C. parvum* burden in stools of infected mice. (F) Quantification of *C. parvum* (mean ± SEM) burden in ileum tissues on day 14 of experimental protocol (*N* = 5 per group). (G) Representative ileal H&E histology from uninfected (nourished or dPD) and infected mice (dPD + Crypto) at day 7 post-infection (p.i.). *C. parvum* induces ileal epithelial damage (black arrow) and inflammatory cell infiltration (red arrow).

A marked decrease in weight gain was observed following protein-deficient diet before (100.1 ± 0.1337% initial weight) and after infection (98.31 ± 0.5638% initial weight), when compared to the control group (102.8 ± 0.9625% initial weight) (Fig. 1A and B), with significant reductions in the tail (nourished vs. dPD vs. dPD + Crypto, 76.89 ± 1.90mm vs. 71.04 ± 4.17mm vs. 69.46 ± 5.70mm) and body length in dPD groups, with or without infection when compared to their controls (Fig. 1B, C and D). Sustained *C. parvum* oocyst shedding was seen with reductions in the shedding of *Cryptosporidium in* feces and the ileal parasite burden over time, but without full clearance (Fig. E).

By histopathology, undernourished mice infected by *C. parvum* exhibited a significant epithelial layer damage, and moderate inflammatory cell infiltration in the ileum (Fig. 1F), resulting in higher histopathologic scores compared to uninfected mice (*p* < 0.05).

**Fig. 2:**
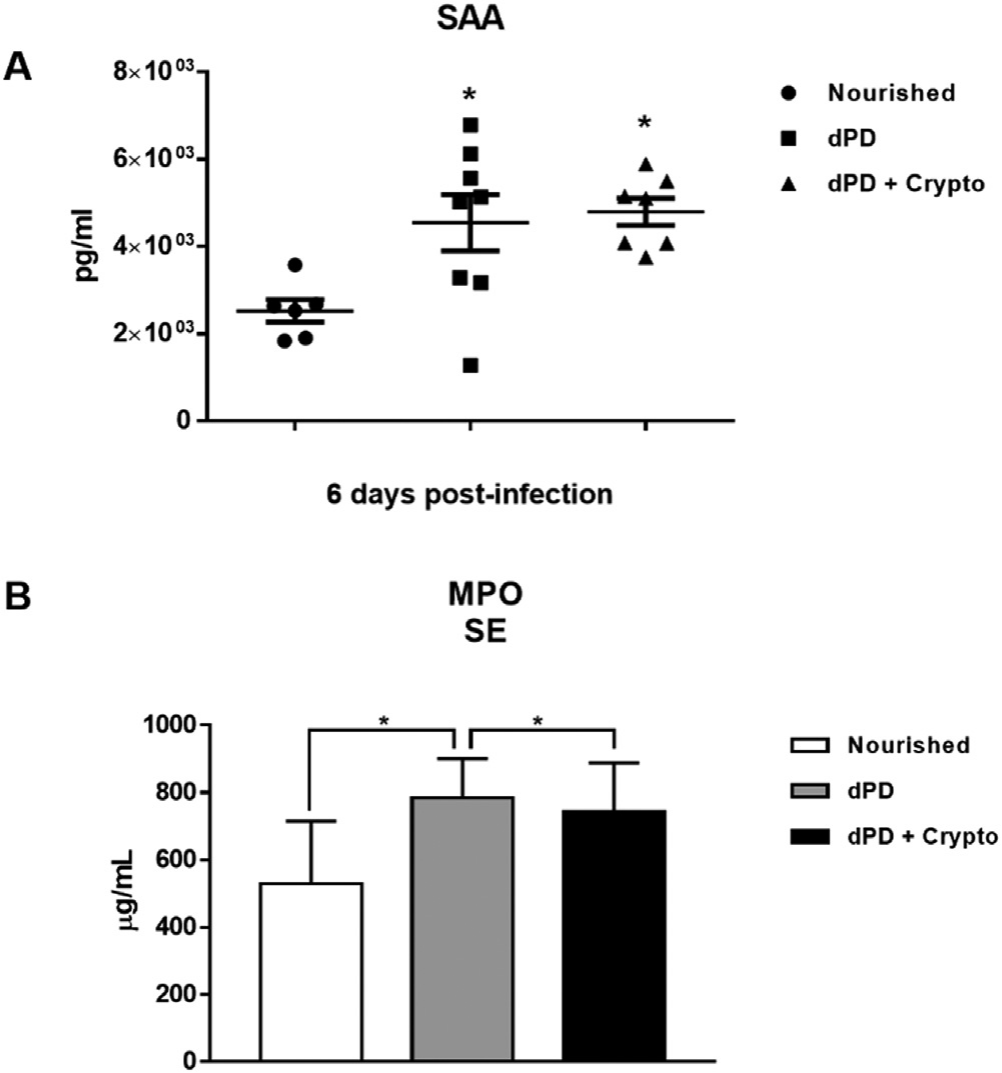
*C. parvum* infection promotes systemic inflammation increasing plasma SAA and MPO in protein-deficient mice. Levels of (A) SAA and (B) MPO (mean ± SEM) measured by ELISA in plasma of uninfected (nourished or dPD; *N* = 7) and infected (dPD + Crypto; *N* = 7) C57Bl/6 mice on day 14 of the experimental protocol. *p < 0.05.

Serum levels of SAA and MPO were increased in animals that received a protein-deficient diet before (4544 ± 644.3pg/ml and 784 ± 47.62μg/ml) and after infection (4792 ± 309.5 and 742.1 ± 55.11) when compared to the uninfected nourished mice (2526 ± 258.1pg/ml and 527.5 ± 70.86μg/ml) (Fig. 2A and 2B).

Undernutrition and *C. parvum* infection led to marked parasite burden, growth, and weight impairment with intestinal/systemic inflammation. SAA and MPO serum levels were significantly higher after dPD and *Cryptosporidium* infection compared to uninfected nourished controls, confirming systemic inflammation.

**Fig. 3:**
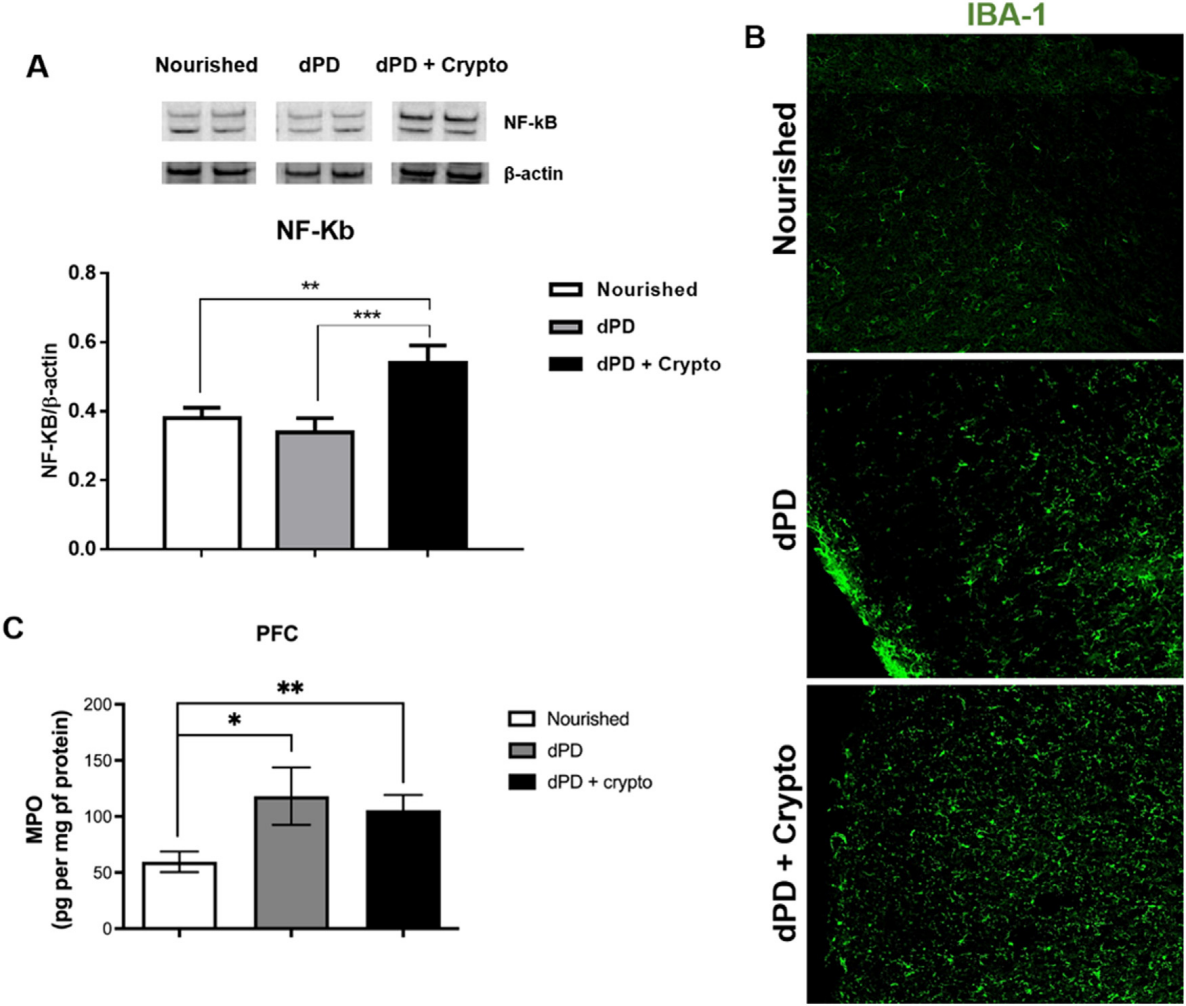
*C. parvum* infection results in inflammation in the prefrontal cortex of mice. (A) Representative western blots of NF-kB and β-actin levels in prefrontal cortex tissues from C57BL/6 mice uninfected (nourished or dPD; *N* = 5) and infected (dPD + Crypto; *N* = 5) on day 14 of the experimental protocol. Bars represent (mean ± SEM) the quantification of western blot bands. #*p* < 0.03. (B) Photomicrographs of Iba-1 (green) immunostaining in prefrontal cortex tissues from C57BL/6 mice uninfected (nourished or dPD) and infected (dPD + Crypto) on day 14 of the experimental protocol. (C) MPO levels (mean ± SEM) measured by ELISA in prefrontal cortex tissues from uninfected (nourished or dPD; *N* = 7) and infected (dPD + Crypto; *N* = 7) C57Bl/6 mice on day 14 of the experimental protocol. *p < 0.05, **p < 0.01 and ***p < 0.001. (For interpretation of the references to color in this figure legend, the reader is referred to the web version of this article.)

Increased NF-kB expression and increased Iba-1 immunolabeling were seen in the prefrontal cortex of undernourished and infected mice when compared to the unchallenged mice (Fig. 3A and B). Higher MPO levels were also seen in the mouse prefrontal cortex after the protein-deficient diet (118.1 ± 25.6pg/mg protein) and *C. parvum* infection (105.7 ± 13.53pg/mg protein), when compared to the control group (59.59 ± 9.166pg/mg protein) (Fig. 3C).

## Discussion

There is accumulating evidence that enteric infections and undernutrition during child development may contribute to cognitive deficits later in life,^7^^,^^8^ but the exact mechanisms of this phenomenon remain to be elucidated. In this context, we show that undernutrition compounded with *Cryptosporidium* infection to increase brain inflammation in our murine model of environmental enteropathy driving brain injury. Modeling early life stress-induced neurologic alterations in animals for investigating their relevant cellular and molecular pathways remains a challenge in the field.

In this study, we show that MPO, a biomarker of enteropathy in children from low and middle-income countries,^16^ is increased in both intestine and brain tissues of weanling undernourished mice with and without cryptosporidial infection. It has been shown that MPO in fecal samples can also be used to compare results in animal models with studies in humans.^23^

Environmental enteropathy is defined as a prolonged intestinal inflammatory condition often seen in children living in poor setting of the developing world exposed to fecal contaminants.^24^ *C. parvum* infections have been recognized to cause intestinal barrier dysfunction^25^ and marked growth faltering in children living under adverse environments, such as poor sanitation and hygiene.^26^^,^^27^

The mouse model of protein deficiency and *C. parvum* infection presented here has been well documented.^28^^,^^29^ This model nicely mimics the so-called vicious cycle of undernutrition and infection in children, as undernutrition suppresses intestinal epithelial turnover and further worsens infection severity and its effects on growth.^30^ This model thus offers an opportune ability to also assess cognitive impairment that may be associated with enteropathy.

Increasing evidence suggests that MPO derived from activated microglia, astrocytes, and neurons could trigger neuropathology.^31^ Further, it has been shown that neutrophil infiltration and myeloperoxidase can disrupt the blood-brain barrier.^32^ In this study, we consistently observed increased activation of proinflammatory pathways in the prefrontal cortex of PD mice, with further activation of NF-kB and Iba-1 with cryptosporidial infection. In addition, Iba-1, a marker of activation in microglia and a major regulator of inflammatory responses in the brain,^31^ was also increased with infection as was NF-kB. Interestingly, in that context of inflammation, MPO was increased in both intestinal and brain tissues, which could drive chronic inflammation.

A recent study suggested that neutrophils and neutrophil extracellular traps are key sources of MPO in the brain during Alzheimer's Disease (AD), which may represent an important mechanism through which blood brain barrier inflammation influences oxidative stress in AD.^33^ We speculate that repeated episodes of enteric infections in the early development of children may induce and sustain MPO levels in the brain, from either gut neutrophils or brain cells, such as activated microglia and neurons. This activation could potentially lead to neuronal injury and cognitive impairment later in life.

Besides increases in MPO in both gut and prefrontal cortex with protein malnutrition in our model, we see further increases in Iba-1 and NF-kB expression levels in the prefrontal cortex of *C. parvum* infections in these mice. These findings suggest that there may be a further augmentation of the NF-kB and Iba-1 involvement in the proinflammatory responses when intestinal infection compounds protein malnutrition.

Although cryptosporidium challenge augments prefrontal cortex NF-kB expression, with more conspicuous Iba-1 immunolabeling, MPO levels did not show higher levels between these groups, suggesting that other signaling pathways may be operating in the brain during *C. parvum* infection.

Executive function, a cognitive domain mostly controlled by the prefrontal cortex, has been consistently found to be compromised in children with history of enteric infections and malnutrition early in life, as seen by neuropsychological examinations.^34^^,^^35^

One limitation is that our study explored MPO as a potential biomarker for intestinal-related brain neuroinflammation and did not further address the fine mechanisms and cytokine signaling pathways associated with MPO-driven effects in the brain. To our knowledge, this is the first study to assess the effects of cryptosporidium infection on the prefrontal cortex in a mouse model of environmental enteropathy. We also could not perform cognitive assessments to the experimental mice due to biosafety issues to avoid cryptosporidium dissemination in the behavior room facility.

An early study found that MPO-positive microglia reside in AD amyloid plaques in the APOE4 brains.^36^ Interestingly, APOE4 was associated with better cognitive outcomes in children with heavy burdens of diarrhea early in life.^37^ However, APOE4 may be a risk factor for acquiring AD and other chronic inflammatory-related diseases later in life for children living in poor sanitation conditions.^38^ More studies are warranted to dissect the associations of APOE4 and MPO during the lifespan in brain neuroinflammation under adverse environments.

In conclusion, our exploratory study describes MPO as a potential biomarker for intestinal-brain axis dysfunction that is relevant in the context of enteric infections and undernutrition in our murine models of protein deficiency and cryptosporidium infection. These findings may help to further understand mechanism of cognitive impairment in children living in impoverished settings and identify children at greatest risk and in need of effective interventions to protect their critical early brain development and ultimate cognitive function. More work is needed to better understand the implications of this work for optimal nutritional and antimicrobial interventions.

## Conflicts of interest

The authors declare no conflicts of interest.
